# Inhibition of Axl Promotes the Therapeutic Effect of Targeted Inhibition of the PI3K/Akt Pathway in NRAS Mutant Melanoma Cells

**DOI:** 10.1155/2022/2946929

**Published:** 2022-03-11

**Authors:** Xuejun Gao, Dandan Xue, Jingjing Cheng, Xin Zhang, Xia Cai

**Affiliations:** ^1^Department of Thyroid Surgery, Affiliated Hospital of Qingdao University, Qingdao, China; ^2^Department of Medical Oncology, Affiliated Hospital of Qingdao University, Qingdao, China; ^3^Department of Neurosurgery, Affiliated Hospital of Qingdao University, Qingdao, China; ^4^Department of Plastic Surgery, Affiliated Hospital of Qingdao University, Qingdao, China

## Abstract

Melanoma is a malignant tumor produced by highly aggressive and metastatic melanocytes. NRAS mutation is a relatively common mutation in melanoma cells. Mitogen-activated protein kinase (MAPK) signaling pathway and the PI3K/Akt pathway in melanoma cells are relatively common signaling pathways. In this study, we investigated the effect of inhibition of Axl expression on the targeted inhibition of the PI3K/Akt pathway in NRAS-mutant melanoma cells. In this study, immunohistochemistry and western blot methods were used to detect the expression of Axl and Akt proteins in melanoma cells. Axl inhibitor was added, and it detected the inhibitory efficiency of Akt inhibitor in melanoma cells. Finally, a melanoma mouse model was established, and it detected the proliferation and apoptosis of mouse tumor cells induced by Axl inhibitor and Akt inhibitor. The results showed that Axl and Akt were highly expressed in NRAS-mutant melanoma cells, and stimulation of Axl expression could reduce the inhibitory effect of Akt inhibitor on melanoma cells. The addition of Axl inhibitor can synergistically promote the effect of Akt inhibitor, slow down the proliferation of tumor cells, and induce cell apoptosis. According to the experiment in this study, Axl inhibitor combined with Akt inhibitor has a stronger therapeutic effect on melanoma than Akt inhibitor alone.

## 1. Background

Melanoma is a highly malignant tumor originating from melanocytes. It mostly occurs in the skin but can also be found in the mucous membrane and viscera. Melanoma is highly malignant and can metastasize at an early stage [1]. According to the World Health Organization, about 50,000 people worldwide die of melanoma each year. In recent years, the incidence and mortality of malignant melanoma have been increasing year by year. Compared with other solid tumors, the death age of malignant melanoma is lower [2]. In addition to early surgical resection, malignant melanoma lacks effective treatment and has a poor prognosis. Therefore, the early diagnosis and treatment of malignant melanoma are extremely important. Currently, the treatment of melanoma is mainly divided into two therapeutic mechanisms [3]: (1) targeted therapy: small-molecule inhibitors targeting the mitogen-activated protein kinase (MAPK) signaling pathway; (2) immunotherapy: biological monoclonal antibodies block cytotoxic T lymphocyte-associated antigen-4 (CTLA-4) and programmed cell death protein 1 (PD-1). Both treatments have their own advantages and disadvantages, and molecular targeted therapy is a major research focus. Molecular targeted therapy is a therapeutic method that specifically selects key enzymes involved in the signaling pathway of cell canceration for targeted inhibition according to different types of gene mutations [4]. At present, a variety of targeted inhibitors targeting different signaling pathways have entered preclinical trials [5]. Among them, RAS/MAPK and PI3K/Akt signaling pathways have been more frequently studied [6], so the development of targeted drugs targeting these two pathways has a broad prospect of clinical application.

Abnormal activation of RAS/RAF/MEK/ERK signaling pathway (MAPK signaling pathway) plays a key role in the occurrence and development of melanoma, and genes related to this pathway are also the main mutation sites of melanoma, especially RAF and RAS genes [7]. Studies have found that 70% of melanomas are associated with BRAF and NRAS gene mutations [8], so there are many studies on BRAF- and NRAS-mutant melanoma. Various small-molecule inhibitors have been extensively studied in BRAF-mutated melanoma cells. For example, the BRAF inhibitor encorafenib can be used in combination with the MEK inhibitor binimetinib to treat advanced BRAF^V600E/K^-mutant melanoma [9]. Cobimetinib combined with vemurafenib in melanoma patients with advanced BRAF^V600^ mutations shows good antitumor activity [10]. The study of NRAS mutation is still under development, so this study selected NRAS mutation melanoma as the research target and studied the use of small-molecule inhibitors for NRAS mutation melanoma.

In melanoma, NRAS is the most common type of mutation in the RAS family, which is commonly seen in congenital pigmented nevi but rarely seen in dysplastic nevi [11]. RAS induces cell proliferation, metastasis, and cell survival through the RAF/MEK/ERK pathway. Therefore, the MAPK pathway plays an important role in the occurrence and progression of NRAS mutated melanoma. Studies have shown that while NRAS activates the downstream MAPK pathway, it also regulates the activation of phosphoinositide 3-kinase-serine/threonine protein kinase B (PI3K-Akt) pathway and thus affects the occurrence of cell apoptosis [12]. Therefore, this study chose MAPK pathway and PI3K/Akt pathway to study melanoma.

In the PI3K/Akt pathway, PI3K is activated by extracellular signals of various cytokine receptors, including tyrosine kinase receptor, nontyrosine kinase receptor, and insulin receptor, which promotes the activation of Akt and further activates a variety of downstream effectors. Akt can affect the adhesion and motor ability of tumor cells by regulating the PI3K/Akt pathway, which is of great significance for the invasion and metastasis of malignant melanoma [13]. Therefore, inhibiting the activity of Akt can inhibit the activity of the PI3K/Akt pathway and then inhibit the proliferation of melanoma. Sanchez-Hernandez et al. [14] found that in the absence of BRAF mutation in melanoma cells, increased phosphorylation of Akt resulted in increased PI3K/Akt pathway activity; inhibition of PI3K/Akt/mTOR pathway activity resulted in increased tumor cell death level. Kuzu OF found that [15], in melanoma cells, Akt inhibition by targeting alone was not obvious, but if targeted together with other enzymes, it could synergistically kill melanoma cells and slow down the growth of tumor cells by 90%. Multiple experiments have shown that BEZ235, an inhibitor of the PI3K/Akt pathway, can effectively inhibit the phosphorylation of Akt and has a good inhibitory effect on the growth of tumor cells in esophageal cancer and glioma cells [16, 17]. In canine melanoma, the combination of BEZ235 and MEK inhibitors effectively reduces the survival rate of melanoma cells and inhibits cell growth [18]. In this experiment, we chose to inhibit the combination of Axl and Akt inhibitors to explore the growth and apoptosis of melanoma cells.

Axl is a tyrosine kinase (RTK), a member of the tumor-associated macrophage (TAM) family. It is composed of Tyro-3, Axl, and Mer [19]. GAS6 and ProS are ligands for TAM. The Axl immunoglobulin-like domain binds to the laminin G-like domains of Gas6 to form the Gas6/Axl complex with high affinity, and the Gas6/Axl complex has a biological activity after translation modification. Axl mediates the proliferation of tumor cells, which is dependent on the MAPK/ERK pathway and involves the activation of PI3K. RAS, Twist, and NF-*κ*B are downstream targets of Axl [20]. Therefore, in melanoma cells, Axl can simultaneously regulate the MAPK pathway and PI3K/Akt pathway, providing a broad research idea for the combination of multiple inhibitors in melanoma. For example, in melanoma, the high expression of Axl makes tumor cells resistant to MAPK pathway inhibitors, and the combination of Axl antibody conjugate and BRAF/MEK inhibitor can synergistically inhibit the growth of tumor cells [21]. Receptor tyrosine kinase (RTK), such as Axl, has great application prospects in melanoma.

Therefore, in this study, melanoma cells with NRAS mutation were selected as the research object, and the activity of Axl was stimulated by Gas6 or the expression of Axl was inhibited by inhibitors, so as to explore the effect of Axl expression on PI3K/Akt pathway and the therapeutic effect of Axl expression on melanoma with NRAS mutation.

## 2. Materials and Methods

### 2.1. Cell Culture

Tumor and paracancerous tissues from melanoma patients were collected, cut into 1 mm [3] size, added with trypsin, and digested at 37°C for 10 min. After elution with 10% FBS and centrifugation at 1,000 r/min for 10 min, the supernatant was removed, and then, the precipitation was resuspended with DMEM medium containing FBS. After centrifugation for another 10 min, the precipitates were collected and resuspended with DMEM medium containing FBS. The cell density was adjusted and planted in the culture flask, and the culture medium was changed once in 2D. All the cells and tissues were cultured in an incubator at 37°C and 5% CO_2_. Cells in the GAS6-induced group were added with 100 ng/mL recombinant human GAS6 solution and cultured for 12 h.

Human NRAS-mutant melanoma cell line SK-MEL-2 and paracancerous normal tissue HEM were all from the Affiliated Hospital of Qingdao University. The sources of cells used in this study have obtained the informed consent of patients.

### 2.2. Western Blot Assay

SK-MEL-2 cells or HEM cells were collected, and the total protein was extracted from tissue cells by protein lysate. An appropriate amount of protein was taken and electrophoretically separated with SDS-PAGE adhesive. Cellulose acetate membrane was used for constant pressure membrane transfer, and then, skimmed milk powder was added to shake at room temperature and sealed for 2 h. After washing with PBS twice, primary antibodies such as rabbit anti-human polyclonal Axl antibody (Abnova Corporation) or rabbit anti-human polyclonal Akt antibody (Abnova Corporation) were added, respectively. They were incubated overnight in a refrigerator at 4°C and washed three times with PBS for 10 minutes each. The second antibody (Abnova Corporation) was added and incubated at room temperature for 2 hours. The PBS was washed three times for 10 minutes each time. Finally, ECL luminescence solution was added for chemiluminescence development, and the western blot fluorescence imager was used to take pictures.

### 2.3. RT-qPCR

The melanoma cell and tissue were collected, and total RNA was extracted using TRIzol Kit (Thermo Fisher, Shanghai) for reverse transcription. The cDNA expression level obtained by reverse transcription was detected by QRT-PCR. The reaction conditions of qRT-PCR were as follows: pre-denaturation at 95°C for 30 s, 95°C for 5 s, 60°C for 1 min, and a total of 40 cycles. The relative expression levels of target genes were calculated using the 2^−△△Ct^ method. The primers sequences were as follows: for Axl, GGCAACCCAGGGAATATCACA (forward) and ACACGAAGGTCTGATGTC CCA (reverse); for Akt, GTGCTGGAGGACAATGACTACGG (forward) and AGCAGCCCTGAAAGCAAGGA (forward); for *β*-actin, GTCCTGTGGCATCCA CGAAAC (forward) and GCTCCAACCGACTGCTGTCAG (reverse).

### 2.4. Immunohistochemical

Melanoma tissue and paracancerous tissue were collected and fixed in paraformaldehyde at room temperature, rinsed with PBS for 3 times, and embedded in paraffin and sectioned. The sections were dewaxed with xylene-ethanol solution, followed by sodium citrate buffer (pH 6.0) for antigen repair, and rinsed with PBS for 3 times. They were put in 30% hydrogen peroxide solution and reacted for 30 minutes in dark at room temperature. They were rinsed with PBS for 3 times. They were blocked with 3% BSA at room temperature for 20 min, then, Axl antibody (Abcam) or Akt antibody (Abcam) was added and incubated overnight in the refrigerator at 4°C. They were rinsed with PBS for 3 times, and HRP-labeled secondary antibody (Abcam) was added at the appropriate concentration and incubated at room temperature for 30 min. They were rinsed with PBS 3 times, 5 minutes each time. DAB chromo-developing solution (Solarbio®, Life Science) was added for staining for 2 min, and sections were rinsed with running water. The hematoxylin solution was redyed for 2 min and rinsed with PBS for 15 min. The slides were dehydrated in ethanol, then, 80% glycerin was added to the slides, and the cover glass was sealed. Finally, microscope observation was performed and photographs were taken (magnification: ×200).

### 2.5. MTT Assay

Cells to be measured were taken and cultured in a DMEM complete medium for 24 h for synchronous treatment. The cell density was adjusted, and then, the cells were inoculated on 96-well plates for culture. Each group was added with the corresponding drug and cultured for a period of time. After the cells grew for a period of time, 5 mg/mL MTT (Sigma) solution was added to each well and placed in an incubator for culture at 37°C for 4 h. The supernatant was discarded, cleaned once with PBS, and 150 *μ*L DMSO (Sigma) was added to each well. The absorbance OD value at 490 nm was measured with a microplate analyzer (Molecular Devices, Shanghai).

### 2.6. Flow Cytometry Was Used to Detect the Number of Early-Regulated Deaths

The cells to be tested were placed in the well plate and then cultured with drugs at the condition of 5% CO_2_ and 37°C for 24 h. After the trypsin (without EDTA) was added, the cells were washed with PBS for 3 times, centrifuged at 1,500 rpm for 5 min, and the supernatant was removed. The procedure was performed according to the instructions of the Annexin V-FITC/PI Apoptosis Assay Kit (ImmunoChemistry, USA). The cells were resuspended by adding 100 *μ*L × binding buffer, 5 *μ*L FITC-Annexin V, and 10 *μ*L propidium iodide, and reacted at darkroom temperature for 15 min. Then, 400 *μ*L × binding buffer was added, and the apoptosis rate of apoptotic cells was detected by flow cytometry.

### 2.7. Cell Clone Formation Experiment

Melanoma cells were digested and dispersed with 0.25% trypsin and cultured in a DMEM medium with 10% fetal bovine serum. NVP-BEZ235 (AmyJet Scientific), RXDX-106 (CEP-40783, Selleck), and GAS6 were added into the medium to grow clone cells. The dishes were washed twice with PBS and fixed with paraformaldehyde for 15 minutes. The fixed solution was removed and stained with crystal violet. The stain plate was rinsed with PBS and allowed to dry at room temperature. Images of the Petri dishes were taken under an inverted light microscope.

### 2.8. Mouse Melanoma Model

Female BALB/c nude mice were purchased from Beijing Vital River Laboratory Animal Technology Co., Ltd. All the animals used in this experiment have been approved by the Ethics Committee of the Affiliated Hospital of Qingdao University. RAS melanoma cell lines were obtained from Tyr: NRAS^Q61K^ transgenic mice, and then, these cell lines were inoculated into the back of female BALB/C nude mice to establish NRAS-mutant melanoma model. Mice were randomly divided into four groups, which were, respectively, injected with normal saline, BEZ235, RXDX-106 (Selleck), and the combination of two drugs. The tumor growth of mice was observed every day. After 28 days, the mice were anesthetized and killed, and the tumor tissue was surgically removed. Then, the mouse melanoma SBcl2 cell line was taken for detection.

### 2.9. Statistical Analysis

SPSS 25.0 software was used for data analysis in this experiment. The *t*-test was used for comparison between the two groups, and the results were expressed as mean ± standard deviation ($\overset{‾}{x}\pm$*s*). *p* < 0.05 was considered statistically significant. The data were processed by GraphPad 8.0 software and presented in the form of charts.

## 3. Results and Discussion

### 3.1. Expressions of Axl in NRAS-Mutant Melanoma Tissues and Cell Lines

**Figure 1:**
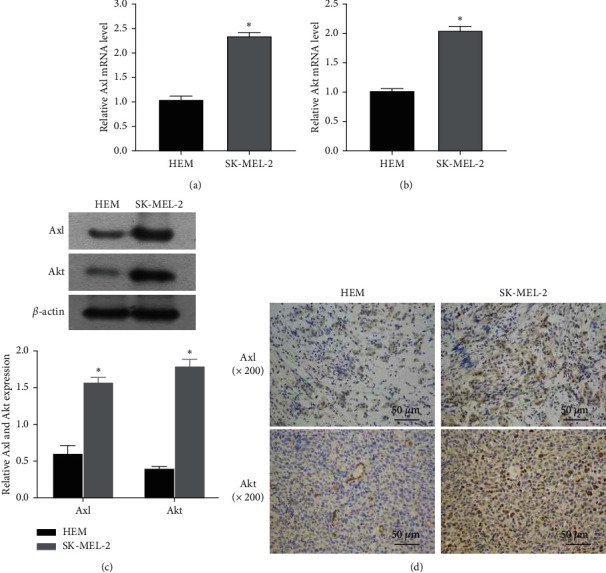
The expressions of Axl and Akt in mutant melanoma cells were detected. (a, b) The expressions of Axl and Akt in mutant melanoma cells were detected by RT-PCR. (c) The expressions of Axl and Akt in melanoma cells were detected by WB. (d) The expressions of Axl and Akt in melanoma tissues were detected by IHC. Compared with the control group, *p* < 0.05, and the difference was statistically significant.

Previous experiments have shown that Axl is expressed in a variety of cancer cells, but there are few studies on melanoma. In this study, we focused on the role of Axl in melanoma cells in order to adopt more effective treatment methods. First, the expressions of Axl and Akt in melanoma tissues were detected by RT-PCR. Both Axl and Akt were highly expressed in melanoma cells, which were significantly higher than that in normal cells (Figures 1(a) and 1(b)). We further selected NRAS-mutated melanoma tissues and used WB to detect the expressions of Axl and Akt in NRAS-mutated melanoma tissues. The results showed that the expressions of Axl and Akt in NRAS-mutated melanoma tissues were higher than that in paracancerous normal tissues (Figure 1(c)). The expressions of Axl and Akt in melanoma cells were detected by IHC. In most melanoma tissues, the expression of Axl was high in the cytoplasm but lower in the paracancerous tissues than in the melanoma tissues. Akt was highly expressed in both the cytoplasm and nucleus of melanoma cells (Figure 1(d)). These results indicate that both Axl and Akt may promote the growth and proliferation of NRAS-mutated melanoma tissue, and it is speculated that there is a synergistic effect between the two.

### 3.2. Activation of Axl Reduces the Efficiency of Akt Inhibitor and Promotes Cell Growth

**Figure 2:**
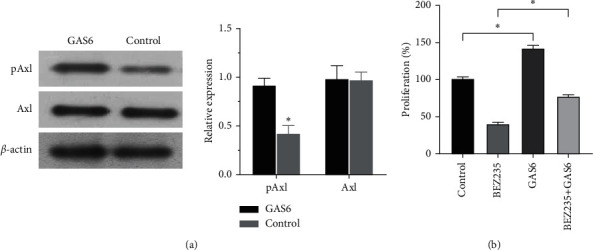
GAS6 stimulated melanoma tissue to detect the effect of Axl activation on the inhibitory efficiency of Akt inhibitor. (a) GAS6 stimulated melanoma tissue, and WB detected Axl activation. (b) Cell proliferation was detected by MTT to determine the effect of Akt inhibitors. Compared with the control group, *p* < 0.05, and the difference was statistically significant.

Previous studies have shown that Ras is the downstream target gene of Axl. As a ligand of TAM, GAS6 can activate Axl when combined with Axl and promote the proliferation of tumor cells. Therefore, GAS6 was selected to stimulate melanoma cells, WB was used to detect the phosphorylation level of Axl, and MTT was used to detect the proliferation of melanoma cells after GAS6 stimulation. The results showed that after GAS6 stimulation, the phosphorylation of Axl in cells was significantly increased, and pAxl activity was increased while the expression remained unchanged (Figure 2(a)). We continued to study the effect of Axl on Akt inhibitors. Melanoma cells with NRAS mutation were treated with GAS6 stimulation and Akt inhibitor (BEZ235) for induction culture, respectively, and cell proliferation was detected by cell cloning assay. Compared with the control group, cell proliferation was reduced after the addition of BEZ235. However, after the stimulation of GAS6, cell proliferation was increased and the inhibitory effect of the Akt inhibitor was reduced (Figure 2(b)). This suggests that the activated expression of Axl can reduce the inhibitory efficiency of Akt inhibitors and promote the proliferation of melanoma cells.

### 3.3. Inhibition of Axl Promotes Apoptosis of NRAS-Mutant Cells Induced by Akt Inhibitors

**Figure 3:**
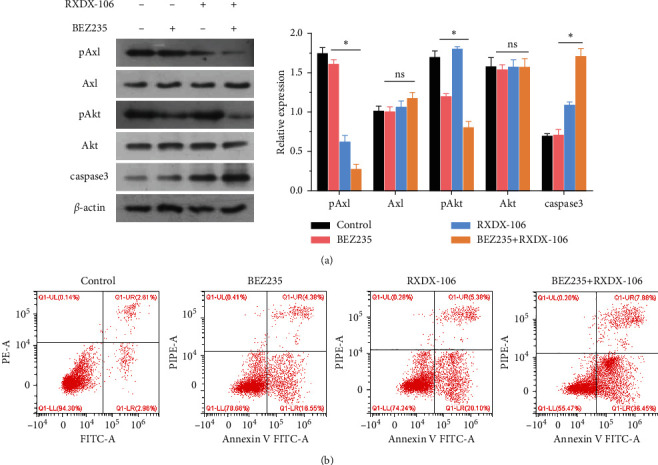
Akt inhibitor BEZ235 was added to determine the apoptotic ability of cells. (a) The phosphorylation activities of Axl and Akt and the activity of apoptotic factor caspase-3 were detected by WB. (b) The apoptosis rate was detected by flow cytometry. Compared with the control group, *p* < 0.05, and the difference was statistically significant.

Since Axl can promote the proliferation of melanoma cells, this study chose to add Axl inhibitors to study the effect of Axl inhibitors on the apoptosis of NRAS-mutant melanoma cells. Axl inhibitor (RXDX-106) and Akt inhibitor (BEZ235) were added into NRAS-mutant melanoma cells, respectively. The activities of phosphorylated Axl, Akt, and apoptosis signaling factor caspase-3 were detected by WB. The results showed (Figure 3(a)) that the activities of pAxl and pAkt decreased after the addition of the corresponding inhibitors, respectively, and the activities of pAxl and pAkt were the lowest in the group with the coinduction culture of the two inhibitors. Compared with the control group, the activity of caspase-3 increased with the addition of inhibitor, and the activity of caspase-3 was the strongest in the group cocultured with RXDX-106 and BEZ235. Cell apoptosis was further detected by flow cytometry. After the addition of inhibitors, the cell apoptosis rate increased, and the cell apoptosis rate was the highest in the group with the addition of two inhibitors (Figure 3(b)). Therefore, inhibition of Axl can synergistically increase the inhibitory effect of the Akt inhibitor and promote cell apoptosis.

### 3.4. Axl Inhibitors Enhance the Efficacy of PI3K/Akt Pathway Targeted Therapy In Vivo

**Figure 4:**
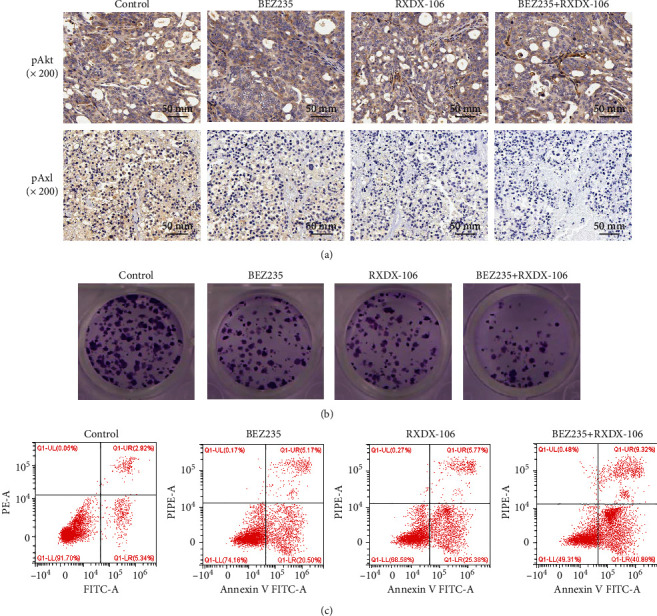
The two inhibitors were used together to determine the growth and apoptosis of tumor cells. (a) The expressions of pAkt and pAxl in mouse transplanted tumors were detected by IHC. (b) Cell cloning was used to examine the proliferation of tumor cells. (c) Apoptosis was detected by flow cytometry.

A mouse model of NRAS-mutated melanoma was established. Axl inhibitor and Akt inhibitor were injected to observe the effects of two inhibitors on the growth and apoptosis of melanoma cells in vivo. First, the IHC was used to examine the expressions of phosphorylated Akt and Axl in the transplanted tumor. The results showed that the addition of RXDX-106 effectively inhibited pAxl, while the addition of BEZ235 effectively inhibited pAkt, and the combination of the two inhibitors further enhanced the inhibition effect. The pAxl and pAkt were the weakest in the RXDX-106 + BEZ235 inhibitor group (Figure 4(a)). Then, cell cloning was used to detect the proliferation of tumor cells in different inhibitory groups. The results showed that compared with the control group, the cell proliferation was significantly decreased in the RXDX-106 and BEZ235 inhibitory groups, and the cell proliferation efficiency was the lowest in the two inhibitor groups (Figure 4(b)). Finally, the apoptosis rate was detected by flow cytometry. With the addition of inhibitors, the apoptosis rate of tumor cells increased. When RXDX-106 and BEZ235 were added at the same time, the cell apoptosis rate was significantly increased (Figure 4(c)). Both RXDX-106 and BEZ235 can effectively inhibit the activity of Axl and Akt and inhibit the growth of melanoma cells. However, the combination of the two inhibitors can more effectively inhibit pAxl and pAkt, and the effect of inhibiting tumor cell proliferation and promoting cell apoptosis is greater than that of one of the inhibitors alone.

## 4. Discussion

Melanoma is a skin tumor that easily metastasizes and is difficult to treat. Patients with melanoma can be treated by surgery at an early stage, but patients with metastasis are not sensitive to radiotherapy or chemotherapy [22]. This suggests that targeted drugs targeting various enzymes will play an important role. RAS has a high mutation rate in melanoma, and studies have found that the inactivation of RAS can lead to rapid death of tumor cells and tumor degeneration, [23] and targeted inhibition of NRAS is also an effective treatment method. However, it is a pity that NRAS lacks the binding sites with small-molecule inhibitors, which cannot be directly inhibited. Therefore, for many years, the treatment of melanoma with NRAS mutation has mainly selected targeted inhibition of its related pathway enzyme components. A number of studies have shown that NRAS-mutated melanoma requires the RAS/RAF/MEK/ERK and PI3K/Akt pathways to induce and maintain malignant phenotypes, [24] and the growth of tumor can also be controlled through the interference of this pathway. For example, the MEK inhibitor binimetinib extends survival in melanoma patients with NRAS mutations and is a new treatment option for melanoma patients with NRAS mutations that have failed immunotherapy [25]. Binimetinib, a MEK inhibitor, has shown good efficacy in combination with CDK4 inhibitors [26]. ERK inhibition combined with PI3K/Akt inhibitor is effective in BRAF inhibitor-resistant cell lines and NRAS-mutant cell lines [27].

Therefore, many experiments have shown that in NRAS-mutated melanoma, the combination of multiple groups of inhibitors can effectively inhibit tumor growth. In this study, IHC, WB, and other detection methods were used to prove that Axl and Akt were highly expressed in melanoma cells. Both Axl and PI3K/Akt pathways are involved in the growth of melanoma cells. Activation of Axl by GAS6, MTT, and cell cloning detected an increased proliferation of tumor cells. Compared with the BEZ235 inhibition group, Axl phosphorylation and cell proliferation were increased after Axl activation, and the inhibitory effect was decreased. After the addition of Axl inhibitor, tumor cell apoptosis increased. The combination of BEZ235 and RXDX-106 inhibitor significantly increased the apoptosis rate of melanoma cells. The results showed that the activity of Axl could affect the PI3K/Akt pathway activity. Inhibition of Axl activity can indirectly inhibit the activity of the PI3K/Akt pathway, synergistically promote the effect of PI3K/Akt inhibitor, inhibit the proliferation of tumor cells, and induce cell apoptosis. To further prove the experimental results, a mouse melanoma model was established, and two inhibitors were given to induce culture. The results showed that compared with the other two groups of mice treated with one inhibitor alone, the tumor growth area of the mice cultured with two inhibitor groups was significantly smaller, the tumor growth rate was slowed down, and the cell apoptosis rate was increased. Since both Axl and PI3K/AKT pathways are involved in the development of melanoma with other gene mutations, the combination of Axl inhibitors and PI3K/AKT pathway inhibitors can be considered for the treatment of melanoma with other gene mutations in subsequent studies.

In the treatment of melanoma, the combination of a variety of inhibitors is an important part of the research. The use of MEK inhibitors in NRAS-mutated melanoma cells has been extensively studied, and more novel inhibition methods are needed. This experiment verified that Axl inhibitor, when used together with PI3K/Akt pathway inhibitor, can promote the inhibitory effect of PI3K/Akt pathway inhibitor, slow down the growth and proliferation of tumor cells, increase the rate of apoptosis, and increase the research direction of double inhibition. Compared with the abovementioned combination of dual inhibitors in the treatment of melanoma with gene mutation, the therapeutic effect of this study is obvious, and the combination of drugs is better. There are still some limitations in this study. Due to the limited samples, there are only a few melanoma cell lines with NRAS mutations. If more different kinds of melanoma cell lines with NRAS mutation can be used, this study will be enriched. The RTK family has a large number of members, and the next step is to explore more and more effective therapeutic targets based on tyrosine kinase receptors.

## Data Availability

The datasets used and/or analyzed during the current study are available from the corresponding author on reasonable request.
